# Factors Influencing Non-urgent Emergency Department Visits: A Review of the Causes and Impact

**DOI:** 10.7759/cureus.107384

**Published:** 2026-04-20

**Authors:** Musaab A Alayob, Saira K Azeem, Saba Ali, Mudhaffar Al Farras

**Affiliations:** 1 Emergency Department, Ras Al Khaimah Medical and Health Sciences University, Ras Al Khaimah, ARE; 2 Emergency Department, Ibrahim Bin Hamad Obaidullah Hospital, Ras Al Khaimah, ARE; 3 Emergency Department, Sultan Qaboos University Hospital, Muscat, OMN

**Keywords:** emergency department, healthcare utilization, health services research, nonurgent visits, systematic review

## Abstract

Emergency departments (EDs) provide time-critical care for acute and potentially life-threatening conditions such as myocardial infarction, stroke, sepsis, severe trauma, and acute respiratory failure. However, increasing use of EDs for non-urgent conditions has been widely reported and is associated with operational pressure on emergency care systems. This systematic review examines key factors associated with adult use of EDs for non-urgent conditions and summarizes the reported impact on emergency care delivery and health systems. A structured literature search was conducted using PubMed/MEDLINE (Medical Literature Analysis and Retrieval System Online), Scopus, and Google Scholar to identify English-language studies published between 2004 and 2024. Studies focusing on adult non-urgent ED utilization were selected based on predefined inclusion criteria and synthesized using a narrative approach due to heterogeneity in study designs, healthcare settings, and definitions of non-urgent visits. Across included studies, 44% of non-urgent ED visits were made by individuals aged 16-35 years, while elderly patients (>65 years) accounted for approximately 2-3%. Non-urgent attendance was commonly associated with low health literacy, such as the belief that EDs provide faster care than primary care, misunderstanding that EDs are the only option for uninsured patients, and a lack of awareness of urgent care clinics and limited awareness of alternative care options, such as telehealth alternatives. Geographic disparities, limited after-hours availability, and delays in primary care services significantly influence ED use. Socioeconomic factors, including low income, lack of insurance, and employment constraints, were also frequently reported. Non-urgent ED visits were seen to be associated with increased crowding and resource strain, potentially contributing to longer waiting times for higher-acuity patients and reduced efficiency of emergency care delivery. Reported strategies include expanding after-hours primary care services, improving public education on appropriate healthcare use, and optimizing ED resource management. Future research should prioritize standardized definitions of non-urgent visits and evaluate interventions across diverse healthcare systems.

## Introduction and background

Emergency departments (EDs) are a central component of modern healthcare systems, providing continuous access to time-sensitive medical care for patients with acute and potentially life-threatening conditions, including severe trauma, acute cardiovascular and neurological events, and critical infections [1,2]. Their role as a safety net for urgent care is well established across diverse healthcare settings [3]. However, alongside this essential function, EDs increasingly serve patients presenting conditions that are classified as non-urgent or low acuity [4].

Non-urgent ED utilization has received growing attention in health services research because of its association with operational challenges such as prolonged waiting times, increased staff workload, and inefficiencies in patient flow [5,6]. ED crowding is widely recognized as a multifactorial phenomenon, influenced by hospital bed availability, population aging, increasing chronic disease burden, and limitations in access to community-based healthcare services [7-9]. Within this context, non-urgent visits are best understood as a contributing factor, rather than the sole cause, of ED crowding and system strain [10].

Estimates suggesting that 40-70% of ED visits are non-urgent are frequently cited in the literature [11,12]. However, these figures vary substantially across studies due to differences in triage systems, urgency classification tools such as the Emergency Severity Index (ESI), healthcare system organization, and study methodology [13-15]. This heterogeneity limits direct comparability across settings and underscores the absence of a universally accepted operational definition of non-urgent ED visits.

Definition of non-urgent ED visits

For the purposes of this review, non-urgent ED visits are defined as low-acuity presentations that do not require immediate life-saving intervention and could reasonably be managed in primary care or other non-emergency settings without anticipated adverse clinical outcomes. This includes visits commonly classified as ESI levels 4-5 or equivalent classifications used in the included studies [13,16]. Given the lack of a universally accepted definition, this review accepted multiple established definitions of non-urgent ED visits and synthesized findings comparatively, with explicit acknowledgment of definitional heterogeneity across healthcare systems.

Existing literature indicates that non-urgent ED utilization is associated with multiple interacting factors, which can be broadly categorized into structural, individual, and system-level domains [17,18]. Structural factors include socioeconomic status, insurance coverage, geographic disparities, and availability of primary care services [19,20]. Individual factors encompass patient perceptions, health literacy, prior healthcare experiences, and expectations regarding diagnostic certainty and speed of care [21-23]. System-level factors include limited after-hours primary care access, fragmented care pathways, and perceived barriers within community healthcare settings [24,25]. These factors interact within specific social and healthcare contexts rather than operating independently.

Non-urgent ED visits have been reported to be associated with adverse system-level outcomes, including increased crowding, longer waiting times for higher-acuity patients, and increased pressure on healthcare staff [26-28]. Associations have also been described between ED crowding and delays in care for time-sensitive conditions such as stroke, myocardial infarction, diabetic ketoacidosis, and severe infections [29-32]. These relationships are generally reported as associations, with proposed mechanisms including delays in triage, treatment initiation, ED patient boarding, and increased staff workload, rather than direct causal effects [33,34].

Despite a growing body of literature, important knowledge gaps remain. Findings related to demographic factors such as age and gender are inconsistent across studies [35-37], and evidence from low- and middle-income countries and rural healthcare settings remains limited [38,39]. In addition, heterogeneity in study design and variability in definitions of non-urgent ED visits complicate synthesis and limit the generalizability of conclusions [40]. These limitations highlight the need for a structured and transparent synthesis of existing evidence.

The objective of this systematic review is to synthesize published evidence on factors associated with non-urgent ED utilization among adults, with particular attention to demographic, access-related, socioeconomic, and perceptual determinants. The review also aims to summarize reported system-level impacts of non-urgent ED visits and to identify gaps in the literature to inform future research and healthcare planning.

## Review

Methods

Study Design

This study was conducted as a systematic review with narrative synthesis examining factors associated with non-urgent ED utilization among adults. A narrative synthesis approach was selected due to substantial heterogeneity in study designs, healthcare systems, outcome measures, and definitions of non-urgent ED visits, which precluded quantitative meta-analysis.

Search Strategy

A comprehensive literature search was conducted in the following electronic databases: PubMed/MEDLINE (Medical Literature Analysis and Retrieval System Online), Scopus, and Google Scholar. The search included studies published between January 2004 and December 2024. This timeframe was selected to capture contemporary evidence reflecting changes in emergency care utilization, healthcare access, and primary care models over the past two decades.

Search terms were developed using a combination of Medical Subject Headings (MeSH) and free-text keywords. Core search concepts included: “non-urgent”, “nonurgent”, “emergency department utilization”, “emergency room visits”, “primary care access”, and “emergency department overcrowding”. Boolean operators (AND/OR) were applied to combine terms appropriately. Controlled vocabulary (MeSH terms) was used where applicable (e.g., PubMed/MEDLINE), while free-text searches were applied in databases without controlled indexing, such as Google Scholar. Google Scholar was included to identify relevant studies not indexed in traditional databases, particularly international and health services research. To minimize duplication, records retrieved from Google Scholar were cross-checked against those from other databases, and duplicate citations were removed before screening.

Eligibility Criteria

Inclusion criteria: Studies were included if they: examined adult populations (≥18 years), addressed non-urgent or low-acuity ED visits, investigated factors associated with ED utilization, including demographic, socioeconomic, access-related, behavioral, or system-level determinants, were published in English, and were published between 2004 and 2024

Exclusion criteria: Studies were excluded if they: focused exclusively on pediatric populations, examined urgent or life-threatening ED presentations only, were published before 2004, were not available in full text, or were published in languages other than English. The restriction to English-language publications and the exclusion of studies due to limited full-text access are acknowledged as potential sources of selection and language bias.

Study Selection Process

The study selection process followed Preferred Reporting Items for Systematic Reviews and Meta-Analyses (PRISMA) guidelines. All identified records were collated and duplicates removed. Titles and abstracts were screened for relevance, followed by a full-text review of potentially eligible studies. Study screening and selection were conducted by a single reviewer due to resource constraints. To mitigate potential selection bias, inclusion and exclusion criteria were predefined and applied consistently throughout the screening process. This limitation is acknowledged and considered in the interpretation of findings.

Data Extraction

Data were extracted using a standardized data extraction form, which captured: author and year of publication, country and healthcare setting, study design, population characteristics, definition or classification of non-urgent ED visits, factors associated with non-urgent ED utilization, and key reported outcomes. Data extraction was performed by a single reviewer. The extraction form was not formally pilot-tested, which may introduce extraction bias and is acknowledged as a limitation.

Definition and Handling of Non-Urgent ED Visits

Definitions of non-urgent ED visits varied across included studies and were commonly based on triage systems (e.g., ESI levels 4-5), retrospective clinical assessment, administrative coding, or author-defined criteria. Rather than applying a single operational definition, this review accepted multiple established definitions and synthesized findings comparatively, with explicit acknowledgment of definitional heterogeneity across healthcare systems.

Data Synthesis

Due to heterogeneity in study designs, populations, and outcome measures, quantitative synthesis was not performed. Instead, findings were synthesized using a narrative thematic approach. Studies were grouped into recurring thematic categories, including demographic factors, structural and access-related determinants, socioeconomic factors, behavioral and perceptual influences, and mental health and substance use considerations. Patterns of consistency and divergence across studies were examined with attention to the healthcare context and study design.

Quality Assessment

A formal quality appraisal or risk-of-bias assessment tool was not applied, given the heterogeneity of included study designs and the narrative nature of the synthesis. The absence of formal quality assessment limits the evaluation of evidence strength and is acknowledged as a methodological limitation.

Methodological Limitations

This review has several limitations, including single-reviewer study selection and data extraction, restriction to English-language publications, exclusion of studies due to limited full-text availability, absence of formal risk-of-bias assessment, heterogeneity in definitions of non-urgent ED visits, and predominance of studies from high-income countries. These limitations were considered when interpreting the findings.

Results

Study Selection and Study Characteristics

**Figure 1:**
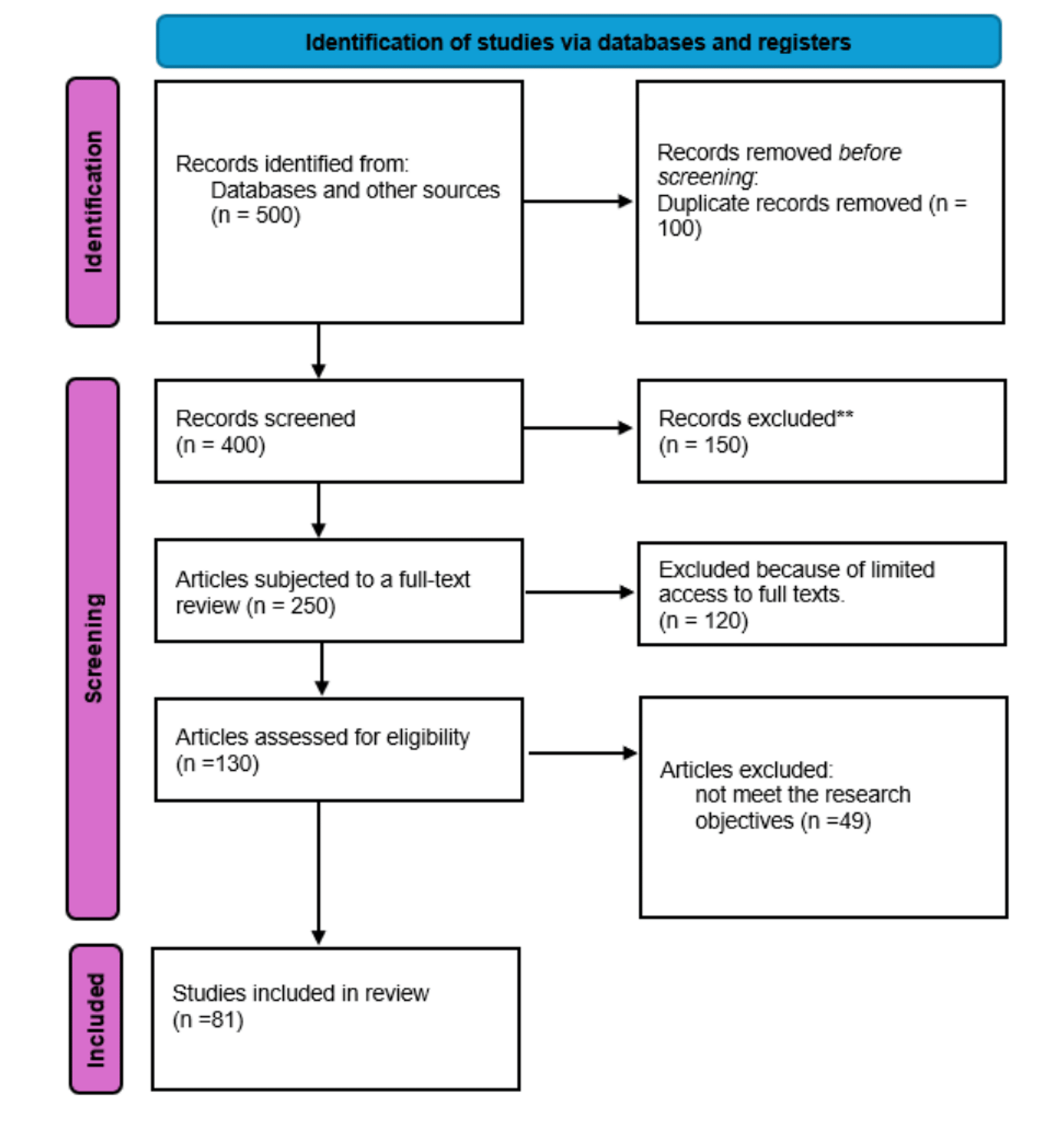
PRISMA flowchart PRISMA: Preferred Reporting Items for Systematic Reviews and Meta-Analyses

A total of 81 studies met the inclusion criteria and were included in the final synthesis (see Appendices). The study selection process is presented in the PRISMA flow diagram (Figure 1). Included studies were published between 2004 and 2024 and examined adult populations presenting to emergency departments for non-urgent or low-acuity conditions.

The included literature comprised a range of study designs, including cross-sectional studies, cohort studies, qualitative studies, policy analyses, and systematic reviews. Primary observational studies constituted most of the included evidence. Systematic reviews were retained to contextualize findings, but were not used to generate pooled estimates, to reduce duplication of evidence. Most studies were conducted in high-income countries, with a predominance of research originating from the United States, followed by studies from Europe, Australia, and Canada. Evidence from middle-income countries was limited, and studies from low-income settings were scarce. Across studies, definitions of non-urgent emergency department visits varied and were commonly based on triage classification systems, administrative coding, retrospective clinical assessment, or author-defined criteria. Study populations, healthcare system structures, and outcome measures also differed substantially. This heterogeneity was considered during data synthesis.

Age-Related Factors Associated with Non-Urgent ED Use

In the included studies, age was frequently examined as a characteristic associated with non-urgent ED utilization, although the direction and magnitude of this association varied across healthcare settings and study designs [12-18]. In many studies, younger adults, commonly defined as individuals aged 18-35 years, represented a substantial proportion of patients presenting with non-urgent conditions to the ED [13,15,17]. The proportion of non-urgent visits attributed to this age group differed widely between studies. Findings related to older adults were less consistent. Some studies reported lower representation of older age groups among non-urgent ED visits, while others identified a notable contribution of older adults, particularly in healthcare systems with limited access to primary or community-based care [14,16,18,41]. These variations are summarized in Table 1 [41-43].

**Table 1:** Age as a factor influencing non-urgent visits

Study	Age Group (years)	% of Non-Urgent ER Visits	Key Findings
Lowthian et al. (2011) [35]	18-35	65%	Younger adults dominate nonurgent ER visits.
Sahwehne et al. (2014) [36]	15-24	Over 50%	Younger adults are the most frequent users of nonurgent ER services.
Hsia and Niedzwiecki (2017) [38]	Below 36	Average age below 36	Average age of nonurgent ER users is below 36.
Alhojelan et al. (2024) [43]	20-34	Majority	Younger adults make up the majority of nonurgent visits.
Coster et al. (2017) [39]	18-35	Significant portion	Younger adults are a significant portion of nonurgent ER visits.
Unwin et al. (2016) [37]	15-24	Most frequent	Most frequent users of nonurgent ER services are younger adults.
Al-Otmy et al. (2020) [40]	40-50	Significantly making nonurgent visits	Middle-aged adults contribute to this trend
Alnasser et al. (2023) [41]	16-35, 36-65, >65	44.2%, 37.7%, 2.3%	Older adults use ER services less for nonurgent

Overall, the relationship between age and non-urgent ED utilization demonstrated considerable heterogeneity across studies [12-18]. Age was therefore identified as an associated demographic characteristic, rather than a consistent or independent determinant, of non-urgent emergency department use.

Gender-Related Factors Associated with Non-Urgent ED Use

Gender was examined as a factor associated with non-urgent ED utilization in many studies, with inconsistent findings across healthcare settings [19-24]. Several studies reported a higher proportion of male patients among non-urgent ED attenders [19,21], while others identified female patients as more frequently represented [20,22]. Several studies reported no significant gender difference in non-urgent ED use [23,24]. The proportion of non-urgent ED visits attributed to each gender varied widely depending on study population, healthcare system structure, and urgency classification method [40-44]. These gender-related patterns are summarized in Table 2.

**Table 2:** Gender as a factor influencing non-urgent ED visits.

Study	Gender Distribution	Key Findings
Sahwehne et al. (2014) [36]	66.6% men, 33.4% women	Men, particularly younger men, were overrepresented in nonurgent ER visits.
Alnahari and A’aqoulah (2024) [42]	56% men, 44% women	Men were the majority of nonurgent ER visitors.
Hsia and Niedzwiecki (2017) [38]	52% women, 48% men	Women were slightly more likely to visit ERs for nonurgent care.
Al-Otmy et al. (2020) [40]	54.8% women, 45.2% men	Women were more likely to use ERs for nonurgent care.
Alnasser et al. (2023) [41]	63.2% women, 36.8% men	A significant majority of nonurgent ER visits were made by women.
Alhojelan et al. (2024) [43]	42.4% men, 40% women	Gender distribution was nearly equal, with no significant influence on nonurgent ER visits.

Overall, gender-related findings demonstrated substantial heterogeneity, and gender was identified as an associated characteristic, rather than a uniform determinant, of non-urgent ED utilization [19-24].

Access to Healthcare and Primary Care-Related Factors Associated with Non-Urgent ED Use

Access-related factors were commonly reported as associated with non-urgent ED utilization across the included studies [34-41]. Limited availability of primary care services, restricted after-hours access, and difficulties obtaining timely appointments were frequently reported among non-urgent ED attenders [35,37,39]. Several studies described higher proportions of non-urgent ED visits among individuals without an established primary care provider or those relying on emergency departments as their usual source of care [34,36,40]. Geographic access barriers, including residence in areas with limited healthcare infrastructure, were also reported in some studies [38,41].

Access-related findings varied substantially across healthcare systems, reflecting differences in primary care organization and service availability [34,41-45]. Access to healthcare was therefore identified as an associated system-level factor, rather than a uniform determinant, of non-urgent ED utilization [46-50]. Articles addressing access to healthcare as an associated factor are shown in Table 3. 

**Table 3:** Access to primary healthcare as a factor influencing non-urgent ED visits

Factor	Study	Key Findings
Geographic Barriers	Cyr et al. (2019) [46]; Simonet (2009) [47]	Rural and underserved urban areas face limited access to healthcare, leading to nonurgent ER visits.
	Coombs et al. (2022) [48]	Under-resourced neighborhoods in urban areas also contribute to nonurgent ER use.
After-Hours Care	Afleck et al. (2013) [17]	Lack of after-hours care drives patients to seek nonurgent ER care.
Primary Care Wait Times	Uscher-Pines et al. (2013) [49]	Long waiting times for primary care led patients to use the ER for quicker access to care.
Perception of ER as Comprehensive	Koziol-McLain et al. (2000) [50]	ER is perceived as a comprehensive care option, influencing patients to bypass primary care providers.

Socioeconomic Factors Associated with Non-Urgent ED Use

Overall, socioeconomic findings demonstrated considerable heterogeneity across studies [25-33]. Socioeconomic status was therefore identified as an associated contextual factor, rather than a consistent or independent determinant, of non-urgent ED utilization.

Socioeconomic characteristics were frequently reported as associated with non-urgent ED utilization across the included studies [25-33]. Lower income levels, lack of health insurance or underinsurance, and unstable employment were commonly observed among populations presenting with non-urgent conditions to the emergency department [26,28,31].

Several studies identified higher proportions of non-urgent ED visits among individuals from lower socioeconomic backgrounds, particularly in healthcare systems where financial barriers limited access to primary or outpatient care [25,27,30]. Other studies reported more variable associations, influenced by national healthcare financing models and availability of publicly funded services [29,32]. Educational attainment was also examined in multiple studies. Lower levels of formal education were frequently reported among non-urgent ED attenders, although the strength of this association varied across study populations and urgency classification methods [26,33, 51-59], as shown in Table 3. 

**Table 4:** Socioeconomic factors influencing non-urgent ED visits

Factor	Study	Key Findings
Income and Financial Barriers	Afleck et al. (2013) [17]; Ratnapradipa et al. (2023) [51]	Lower income and financial strain lead to delays in care, causing patients to use ERs for nonurgent issues
Employment Status	Carret et al. (2009) [54]; Hodgins and Wuest (2007) [55]	Low-wage jobs and unstable employment lead individuals to rely on ERs for nonurgent care due to lack of time and access.
Education and Health Literacy	Griffey et al. (2014) [56]; Vogel et al. (2019) [57]	Low health literacy leads to higher nonurgent ER use due to misunderstanding of symptoms and lack of knowledge of alternative options.
Insurance Coverage	Weinick et al. (2010) [26]; Carret et al. (2009) [54]	Uninsured or underinsured patients are more likely to use ERs for nonurgent care due to concerns about healthcare costs.
Mental health and substance use disorders	Henderson et al., (2019) [58]; Moe et al., (2022) [59]	Substance use disorders and mental health conditions often rely on ER for nonurgent care

Mental Health and Substance Use-Related Factors Associated with Non-Urgent ED Use

Mental health conditions and substance use were examined in several of the included studies and were reported as associated with non-urgent ED utilization, although the extent of this association varied across study populations and healthcare settings [42-48]. Studies consistently reported higher proportions of non-urgent ED visits among individuals with anxiety disorders, depression, and other common mental health conditions, particularly in settings where access to outpatient mental health services was limited [43,45,47]. Substance use, including alcohol and non-medical drug use, was also reported as a frequent characteristic among non-urgent ED attenders in multiple studies [44,46,48]. The prevalence of substance use-related non-urgent visits varied across studies, reflecting differences in study design, population characteristics, and classification of visit urgency.

Mental health and substance use-related findings demonstrated substantial heterogeneity, with variation influenced by healthcare system structure, availability of behavioral health services, and social context [42-48]. These factors were therefore identified as associated clinical and behavioral characteristics, rather than independent determinants, of non-urgent ED utilization across the included literature.

System-Level Outcomes Associated with Non-Urgent ED Use

Several included studies examined system-level outcomes associated with non-urgent ED utilization, particularly indicators related to crowding, waiting times, and workload [49-56]. Non-urgent ED visits were frequently reported alongside longer waiting times, increased patient volume, and higher staff workload, although the magnitude of these associations varied across healthcare settings [50,52,54]. Multiple studies reported associations between higher proportions of non-urgent visits and ED crowding, measured using indicators such as length of stay, occupancy rates, or delays in patient flow [49,51,53]. In some settings, non-urgent visits were described as occurring concurrently with delays in care for higher-acuity patients, while other studies emphasized that crowding reflected multifactorial system pressures, including inpatient bed availability and staffing constraints [55,56].

Overall, system-level findings demonstrated substantial heterogeneity, with variations influenced by healthcare system structure, case-mix, and methods used to assess crowding and delay. System-level outcomes were therefore reported as associated operational challenges, rather than direct consequences, of non-urgent ED utilization across the included studies [49-59].

Impact of Non-urgent ED Visits on Healthcare Systems

Non-urgent ED visits were associated with multiple system-level consequences, including overcrowding, prolonged waiting times, increased healthcare costs, and increased provider workload [59-64]. Several studies reported that high volumes of nonurgent cases contributed to congestion within EDs, potentially delaying care for patients with time-sensitive conditions such as myocardial infarction and stroke [61-65].

Treating non-urgent conditions in emergency settings was consistently shown to be more costly than management in primary or urgent care settings [26,64]. The diversion of ED resources toward nonurgent care was also associated with reduced quality of care for critically ill patients, increased provider workload, and higher levels of staff burnout [11,66-69]. From the patient perspective, nonurgent ED visits were linked to fragmented continuity of care and psychological stress related to prolonged waiting times and extensive diagnostic testing [70-75]. Together, these findings highlight the broader operational and experiential implications of nonurgent ED utilization for healthcare systems and patient experience.

In addition, several studies highlighted that fragmentation between EDs, primary care, and community-based services contributes to inappropriate ED utilization and limits continuity of care [76-78]. Variability in triage systems, urgency classification tools, and administrative coding practices further complicates the interpretation and comparison of nonurgent ED utilization across studies and healthcare systems [79,80]. The different impacts of non-urgent visits on the healthcare system are shown in Table 5.

**Table 5:** The impact of non-urgent visits

Impact	Key Findings	Citations
Overcrowding in ERs	Nonurgent visits contribute significantly to ER overcrowding, leading to increased wait times for critical patients and delayed diagnoses.	Hoot and Aronsky (2008) [61]; He et al. (2011) [60]; Asplin et al. (2003) [63]
Financial Burden on Systems	Treating nonurgent conditions in the ER is much more expensive than treating them in primary care, resulting in higher costs for both healthcare systems and patients.	Weinick et al. (2010) [26]; Flores-Mateo et al. (2012) [64]; Daly (2022) [65]
Reduced Quality of Care	The diversion of resources to nonurgent cases diminishes the quality of care for critical patients, leading to higher morbidity and mortality rates.	Lu et al (2021) [68]; Bernstein et al. (2009) [69]
Misuse of ER Resources	Nonurgent cases often receive unnecessary diagnostic tests, leading to misallocated resources and longer wait times for patients with critical needs.	Durand et al. (2012) [31]; William et al. (2019) [32]
Fragmented Continuity of Care	Nonurgent visits disrupt continuity of care, particularly for patients with chronic conditions, leading to poor long-term health outcomes.	Weinick et al. (2010) [26]
Psychological Impact on Patients	Nonurgent visits to the ER increase patient anxiety and frustration, especially due to long wait times and unnecessary diagnostic tests.	Sun et al (2013) [71]; Hunter (2013) [73]; Richardson (2006) [70]

Finally, the absence of standardized definitions and consistent outcome measures was repeatedly identified as a key methodological limitation in the literature, hindering robust synthesis and evaluation of interventions aimed at reducing nonurgent ED visits [72,81-83].

Discussion

Recommendations

Based on the findings of this review, several evidence-informed recommendations may be considered to address non-urgent ED utilization. Given the multifactorial nature of non-urgent ED visits, interventions should be multidimensional and context-specific, rather than relying on a single strategy. Improving access to timely primary and urgent care services may help reduce reliance on EDs for non-urgent conditions, particularly in underserved urban and rural settings. Expanding after-hours primary care availability and strengthening referral pathways could provide viable alternatives for patients seeking care outside regular clinic hours. Enhancing coordination between emergency departments, primary care providers, and community health services may also support more appropriate healthcare utilization. Targeted patient education initiatives aimed at improving health literacy and awareness of available healthcare options should be considered. Educational interventions that help individuals assess symptom urgency and navigate healthcare services may reduce uncertainty and unnecessary ED attendance, especially among younger adults and socioeconomically disadvantaged populations.

From a system perspective, healthcare planners should consider strategies that support appropriate triage and resource allocation within emergency departments, ensuring that critical cases receive timely care while nonurgent presentations are managed efficiently. Integrating mental health and substance use services within community and outpatient settings may further reduce recurrent nonurgent ED visits among vulnerable populations. Finally, future research should prioritize the use of standardized definitions of nonurgent ED visits and adopt robust methodological designs to improve comparability across studies. Evaluating the effectiveness of targeted interventions across different healthcare systems and populations will be essential to inform sustainable and evidence-based solutions.

Limitations

This review has several limitations that should be considered when interpreting the findings. First, the review was limited to studies published in English, which may have resulted in the exclusion of relevant evidence from non-English-language publications. This language restriction may introduce selection bias and limit the global generalizability of the findings. Second, the review included studies published over a broad time frame (2004-2024). While this approach allowed for a comprehensive overview of literature, changes in healthcare systems, ED utilization patterns, and access to alternative care models over time may limit the comparability of findings across older and more recent studies. Third, substantial heterogeneity was present across the included studies with respect to study design, populations, healthcare settings, and definitions of non-urgent ED visits. Variability in triage systems and urgency classifications may have influenced prevalence estimates and contributed to inconsistencies in reported associations. As a result, direct comparison across studies should be interpreted with caution. Fourth, the review relied primarily on observational studies, which limits the ability to draw causal inferences. Associations identified between demographic, socioeconomic, and access-related factors and non-urgent ED use should not be interpreted as evidence of causation. In addition, no formal meta-analysis was conducted due to methodological heterogeneity among the included studies. Fifth, although a standardized data extraction approach was used, a formal risk-of-bias or quality assessment tool was not applied across all included studies. This limits the ability to evaluate the relative strength of the evidence and may affect the interpretation of synthesized findings. Finally, the majority of included studies were conducted in high-income countries, particularly the United States. Evidence from low- and middle-income settings was limited, which may restrict the applicability of the findings to healthcare systems with different access models and resource constraints.

## Conclusions

This systematic review synthesizes evidence on factors associated with non-urgent ED utilization among adults, highlighting the complex and multifactorial nature of this phenomenon. The findings indicate that nonurgent ED visits are associated with demographic characteristics, particularly age and gender, as well as structural barriers to healthcare access and socioeconomic conditions, rather than being driven by a single determinant. Patterns of non-urgent ED use varied across studies, reflecting differences in healthcare systems, population characteristics, and definitions of non-urgency. Younger adults were more frequently represented among non-urgent ED users, while findings related to gender were inconsistent across settings, underscoring the influence of contextual and system-level factors.

Limited access to timely primary care, restricted after-hours services, and geographic barriers were repeatedly associated with increased reliance on emergency departments for non-urgent conditions. Socioeconomic disadvantages, lower health literacy, and inadequate insurance coverage further shape healthcare-seeking behavior and contribute to recurrent nonurgent ED use. Across the reviewed literature, nonurgent ED visits were associated with operational challenges, including overcrowding, increased healthcare costs, and strain on emergency care resources. These associations highlight the importance of addressing non-urgent ED utilization within broader health system planning, while recognizing that the evidence is largely observational and heterogeneous in design. Future efforts should focus on improving access to primary and urgent care services, enhancing health literacy, and developing system-level interventions tailored to local healthcare contexts. Further research using standardized definitions and robust methodological approaches is needed to better understand non-urgent ED utilization and to evaluate the effectiveness of targeted interventions across diverse healthcare systems.

## Appendices

**Table 6:** Detailed summary of included studies

Study	Country/Health System	Study Design	Relevance to Study	Key Findings
1. Mistry et al. (2008) [15]	United States	Cross-sectional Study	Examine urgency classification methods for ED visits	Evaluates methods for classifying nonurgent ED visits
2. Afilalo et al. (2022) [76]	Canada	Observational Cohort Study	Examine access to primary care as a factor in ED use	Limited access to primary care increases unplanned ED visits, particularly among elderly patients.
3. Afonso and Lopes (2020) [29]	Portugal	Retrospective Cohort Study	Explores characteristics of high-frequency ED users	High-frequency ED users often have complex medical and social needs that primary care does not fully address.
4. Al-Arifi and Al-Rashdi (2018) [30]	Saudi Arabia	Cross-sectional Study	Investigates accessibility to primary healthcare and its impact on ED	Poor access to primary healthcare correlates with increased ED visits for nonurgent issues.
5. Alhojelan et al. (2024) [43]	Saudi Arabia	Cross-sectional Survey	Analyzes patterns in nonurgent ED visits	Lack of awareness about triage systems and motivations based on convenience drive nonurgent ED visits.
6. Alnahari and A’aqoulah (2024) [42]	Saudi Arabia	Cross-sectional Study	Examines demographic factors influencing ED length of stay	Demographic variables like age and socioeconomic status impact ED visit length, affecting overcrowding.
7. Al-Otmy et al. (2020) [40]	Saudi Arabia	Cross-sectional Study	understanding nonurgent visit motivations	Nonurgent ED visits are associated with convenience and perceived lack of alternatives.
8. O'Cathain et al. (2020) [83]	United Kingdom	Realist Review	Provides details about nonurgent ED visits	Highlights variability in definitions and lack of standardization, limiting comparability and evaluation of interventions
9. Backman et al. (2008) [11]	Sweden	Cross-sectional Study	Compares primary care versus ED for nonurgent visits	Nonurgent ED visits often result from patients’ perception of faster service and better care compared to primary settings.
10. Bakarman and Njaifan (2014) [78]	Saudi Arabia	Cross-sectional Study	Identifies patterns in nonurgent ED visits	Nonurgent ED visits arise due to perceived need for immediate care, irrespective of actual urgency.
11. Bahadori et al. (2020) [8]	Iran	Qualitative descriptive Study	Explores cultural causes of nonurgent ED use	Cultural factors and lack of alternative facilities contribute to high nonurgent ED visit rates in Iran.
12. Berchet (2015) [2]	OECD Countries	Systematic Review	Provides insights into global ED use trends	Identifies socioeconomic and systemic factors influencing ED use across various health systems.
13. Bernstein et al. (2009) [69]	United States	Observational Study	Studies impact of crowding on patient outcomes	ED crowding, exacerbated by nonurgent visits, negatively impacts clinical outcomes for patients.
14. Mckenna et al (2019) [24]	united state	review article	Examine causes, consequences and solution of ED crowding	Highlights that ED crowding is multifactorial and driven by system-level factors, not solely nonurgent visits.
15. Caldwell et al. (2013) [25]	United States	Cross-sectional Analysis	Assesses patient awareness of charges in ED	Financial transparency may deter nonurgent visits, as patients are unaware of cost implications.
16. Carret et al. (2009) [54]	Brazil	Systematic Review	Examines socioeconomic factors affecting ED use	Highlights socioeconomic disparities as key factors for nonurgent ED use in Brazil.
17. Lu et al. (2021) [68]	United States	Observational Study	examine racial/ethical differences in ED wait times.	Minority patients (black and Hispanic) experience longer ED wait time, indicating disparities in timely access to emergency care.
18. Coster et al., (2017) [39]	United Kingdom	Rapid Review	Investigates patient choice between urgent care and ED	Accessibility and patient convenience are significant drivers of ED utilization over urgent care services.
19. Cowling et al. (2014) [28]	England	Cross-sectional Study	Studies the relationship between GP access and ED visits	Limited GP availability directly correlates with increased ED visits for nonurgent issues.
20. Coombs et al. (2022) [48]	United States (Rural Healthcare)	Qualitative Study	Explores healthcare access barriers in rural areas	Rural populations face unique social and cultural barriers that drive nonurgent ED visits.
21. Cyr et al. (2019) [46]	United States	Systematic Literature Review	Studies urban versus rural healthcare access disparities	Limited access to specialty care in rural areas drives patients to seek nonurgent care in EDs.
22. Daly (2022) [65]	United States	Financial Analysis	Assesses economic burden of preventable ED use	Nonurgent visits contribute significantly to healthcare costs, especially when ED visits are preventable.
23. Detollenaere et al. (2014) [27]	Belgium (Flanders)	Explorative Study	Examines socioeconomic influences on ED use	Socioeconomic status strongly influences patients’ choice of ED over primary care during out-of-hours periods.
24. Durand et al. (2012) [31]	France	Mixed-Methods Study	Compares perceptions of ED use among professionals and patients	Nonurgent ED users often perceive themselves as justified, while professionals view these visits as avoidable.
25. Flores-Mateo et al. (2012) [64]	Spain	Systematic Review	Reviews of organizational interventions to reduce ED use	Organizational changes, such as improving GP access, can reduce nonurgent ED visits.
26. Hunter et al. (2013) [73]	United Kingdom	Qualitative Study	Examines patient decision making in seeking urgent and emergency care.	Recursivity and candidacy provide a framework for understanding patient decision-making around emergency care use.
27. Sun et al. (2013) [71]	United States	Retrospective cohort study	Examines impact of ED crowding.	ED crowding is associated with increased mortality and delays in care.
28. Gabil et al. (2020) [9]	Saudi Arabia	Retrospective Analysis	Studies impact of overcrowding with nonurgent visits	Nonurgent visits increase ED mortality risk by diverting resources from critical cases.
29. Gentili et al. (2021) [44]	Italy	Cross-sectional Study	Examine predictors of nonurgent ED access among older adults.	Frail older adults frequently use EDs for nonurgent needs due to lack of accessible alternatives.
30. Gindi et al. (2012) [66]	United state	Cross-sectional Survey	Assesses ER use among working-age adults	Nonurgent ED visits are common among adults who face access barriers during traditional clinic hours.
31. Goodridge and Stempien (2019) [74]	Canada	Qualitative Study	Explores reasons for nonurgent ED visits from older people's perspective	Older adults often cite issues with primary care access and timeliness as reasons for ED visits.
32. Griffey et al. (2014) [56]	United States	Retrospective Cohort Study	Investigates link between health literacy and ED usage	Low health literacy is associated with higher nonurgent ED use due to misunderstandings about care options.
33. Grumbach et al. (2010) [34]	United States	Cross-sectional Analysis	Explores public ED usage and primary care availability	Lack of timely primary care access contributes to nonurgent ED visits, exacerbating overcrowding.
34. Ahlin et al. (2022) [13]	Sweden	Systemic Review	Examines hospital-wide throughput barriers affecting patient flow.	ED crowding is driven by system-level throughput barriers (e.g. delay in admission and discharge), not only demand.
35. He et al. (2011) [60]	Australia	Conceptual Review	Examines ED demand through conceptual models	presents a theoretical framework on factors affecting ED demand, emphasizing primary care accessibility.
36. Van den Heede and Van de Voorde (2016) [82]	Belgium	Systematic Review	Reviews interventions to reduce ED use	Highlights various interventions, including primary care integration, as effective in reducing nonurgent ED visits.
37. Henderson et al. (2020) [58]	United States	Comparative Study	Examines health service usage post discharge	Patients with mental illness use EDs at higher rates post discharge from community mental health centers.
38. Higginson (2012) [3]	United Kingdome	Structured Literature Review	Reviews ED crowding and its implications	Highlights systemic issues that drive ED crowding, such as patient expectations and primary care access issues.
39. Hodgins and Wuest (2007) [55]	Canada	Qualitative Study	Examines urban‒rural differences in ED use for nonurgent cases	Rural areas show higher nonurgent ED use due to fewer accessible primary care facilities.
40. Hoot and Aronsky (2008) [61]	United States	Systematic Review	Investigates causes, effects, and solutions for ED crowding	Identifies causes of nonurgent ED visits, emphasizing health literacy and healthcare access issues.
41. Hsia and Niedzwiecki (2017) [38]	United States	Retrospective analysis	Examines avoidable ED visits and factors influencing them	Discusses factors like insurance and primary care access that influence avoidable ED visits, especially nonurgent cases.
42. Hu et al. (2017) [1]	Taiwan	Retrospective Analysis	Studies predictors for ED revisits	Family factors and perception of urgent care need impact patient revisit rates, even for nonurgent symptoms.
43. Joanna (2015) [21]	Australia	Doctoral Dissertation	Studies demand for ED services and associated patient factors	Finds that patient convenience and lack of primary care access increase nonurgent ED demand in urban areas.
44. Johnson and Winkelman (2011) [62]	United States	Literature Review	Investigates impact of crowding on patient outcomes	ED crowding from nonurgent visits is associated with delayed care and worsened outcomes for high-acuity patients.
45. Alnasser et al. (2023) [41]	Saudi Arabia	Retrospective Chart Review	Analyzes characteristics of nonurgent ED visits	Nonurgent visits are often linked to younger patients and those without prior primary care engagements.
46. Kim and Lee (2022) [23]	South Korea	Retrospective Cohort Study	Examine ED length-of-stay management effects	Effective management systems reduce ED length of stay for nonurgent visits, helping manage overall patient flow.
47. Koziol-McLain et al. (2004) [50]	United States	Qualitative Study	Explores patient reasons for nonurgent ED use	Patients choose EDs due to perceived immediacy of care and difficulty accessing other healthcare settings.
48. Lega and Mengoni (2008) [14]	Italy	Empirical Study	Analyzes patient choices between ED and primary care	Nonurgent ED visits are often driven by convenience factors and perceptions of quality care in ED settings.
49. Leshinski et al. (2023) [19]	Israel	Qualitative Study	Defines and validates criteria for nonurgent ED referrals	Provides insight into standardized criteria for triaging nonurgent cases to improve ED resource allocation.
50. Levesque et al. (2013) [45]	Canada	Conceptual Framework	Examines patient-centered access to healthcare	Proposes a framework for understanding access barriers that contribute to nonurgent ED visits.
51. Lima et al. (2015) [5]	Brazil	Cross-sectional descriptive study	Measures quality of ED care from patient perspective	Satisfaction is influenced by waiting times, perceived need, and lack of primary care access, impacting ED use trends.
52. Lobachova et al. (2014) [67]	United States	Cross-sectional Survey	Assesses perceptions on ED utilization by patients and providers	Highlights differing views between patients (convenience) and providers (resource misuse) on nonurgent ED use.
53. Pines et al. (2011) [6]	International	Observational/Descriptive	Examines characteristics and impact of nonurgent ED visits.	Nonurgent ED use increases patient volume and contributes to service demand, leading to crowding and reduced efficiency in emergency care delivery
54. Lowthian et al. (2011) [35]	Australia	Systematic Review	Studies trends in ED attendance from an Australian ED perspective	Identifies increasing nonurgent ED visits, driven by aging populations and healthcare access challenges.
55. McIntyre et al. (2023) [18]	Canada	Integrative Review	Examine patient-reported reasons for nonurgent ED use	Nonurgent ED visits are often influenced by perceptions of convenience and faster care.
56. McCaig and Nawar (2006) [80]	United States	National Survey Analysis	Analyzes trends in ED usage	Nonurgent ED visits vary significantly across age and insurance status.
57. Uscher-Pines et al. (2013) [49]	United States	Systematic Review	Identifies nonurgent cases suited for other care settings	Many ED visits could be redirected to urgent care centers or retail clinics.
58. Mendes et al. (2021) [12]	Brazil	Comparative Retrospective Cohort Study	Examines ICU wait time impact on ED patients	Extended ED stays for ICU-bound patients affect nonurgent care processing.
59. Morley et al. (2018) [22]	Global	Systematic Review	Assesses causes and solutions for ED crowding	Nonurgent visits are a primary contributor to ED crowding worldwide.
60. Moskop et al. (2009) [4]	United States	Review Article	Reviews of moral implications of ED crowding	Nonurgent visits divert critical resources, raising ethical concerns in care.
61. Moe et al. (2022) [59]	Canada	Retrospective Cohort Study	Focuses on high ED users with substance use	Patients with substance use disorders show high nonurgent ED utilization.
62. Newton et al. (2008) [33]	United States	Meta-analysis	Investigates uninsured adult usage of ED	Uninsured adults frequently rely on ED for nonurgent needs due to lack of alternatives.
63. Nguyen et al. (2018) [52]	United States	Qualitative Focus Group Study	Explores financial strain's effect on ED decisions	Financial stress leads some patients to prefer EDs over primary care for perceived affordability.
64. Nawar et al. (2005) [16]	United States	National Health Statistical Analysis	Analyzes ED visit patterns and delays	ED visit delays are often linked to nonurgent cases that could be handled elsewhere.
65. Ramlakhan et al. (2016) [53]	United Kingdom	Literature Review	Examines collocated primary care and ED effectiveness	Collocated services can reduce nonurgent ED visits by offering immediate primary care access.
66. Rabin et al. (2016) [7]	United States	Systematic Review	Evaluates solutions to reduce ED 'boarding' and crowding	Suggests legislation for redirecting nonurgent cases away from EDs to alleviate crowding.
67. Richardson (2006) [70]	Australia	Retrospective Cohort Study	Examine impact of crowding on patient mortality	ED overcrowding, including nonurgent cases, is associated with increased mortality risk.
68. Tanabe et al. (2007) [20]	United States	Cross-sectional Study	Evaluates Emergency Severity Index (ESI) in ED triage	The ESI tool aids in diverting nonurgent cases from EDs to manage resources better.
69. Sahwehne et al. (2014) [36]	Jordan	Cross-sectional Study	Assesses nonurgent ED visit patterns	Nonurgent ED visits are largely driven by convenience and lack of primary care access.
70. Shahid et al. (2022) [79]	Canada	Prospective Cohort Study	Examine effect of health literacy on ED use	Low health literacy is linked to increased nonurgent ED visits for minor conditions.
71. Simonet (2018) [47]	France	Policy Analysis	Analyzes health system accountability reforms	Health reforms aimed at improving primary care access reduce nonurgent ED visits.
72. Sørup et al. (2013) [81]	Denmark	Systematic Review	Evaluates ED performance and quality measures	Highlights that nonurgent visits reduce ED efficiency and affect quality measures.
73. Street et al. (2018) [75]	Australia	Retrospective comparative Cohort Study	Analyzes frequent ED use by older adults	Older adults often choose EDs for convenience and due to insufficient primary care support.
74. Unwin et al. (2016) [37]	Australia	Patient Perspective cross-sectional Survey	Explores patient perspectives on ED services	Nonurgent ED users cite accessibility and timely service as the main reasons for their visits.
75. Afleck et al. (2013) [17]	Canada	Position statement	Explores overcrowding of ED and benchmarks	ED overcrowding is a public health concern; the adoption of national benchmarks will provide goals
76. Vogel et al. (2019) [57]	United States	Qualitative Meta-synthesis	Summarizes reasons patients prefer ED over primary care	Patients often perceive EDs as more convenient and offer faster care than primary providers.
77. Ratnapradipa et al. (2023) [51]	United States	Cross-sectional Study	Investigate factors for delayed medical care	Patients delay care and use ED for nonurgent issues due to lack of affordable alternatives.
78. Wavetec (2024) [72]	Global	Healthcare Report	Analyzes waiting times in ER and patient satisfaction	Patient satisfaction in ED is impacted by waiting times, especially for nonurgent cases.
79. Weinick et al. (2010) [26]	United States	Comparative study	Evaluates alternative sites for nonurgent cases	Suggests urgent care and retail clinics as alternatives for nonurgent ED cases.
80. William et al. (2010) [32]	Taiwan	Survey based// Cross-sectional Study	Explores patient preference for ED versus other options	Patients prefer ED due to convenience and perceived quality of care, despite nonurgent needs.
81. Owens (2008) [77]	United States	Cross-sectional Analysis	Investigates gender differences in ED use	Nonurgent visits are more common among certain demographics due to convenience and accessibility.
